# The *miR-181* family promotes cell cycle by targeting *CTDSPL*, a phosphatase-like tumor suppressor in uveal melanoma

**DOI:** 10.1186/s13046-018-0679-5

**Published:** 2018-01-30

**Authors:** Leilei Zhang, Xiaoyu He, Fang Li, Hui Pan, Xiaolin Huang, Xuyang Wen, He Zhang, Bin Li, Shengfang Ge, Xiaofang Xu, Renbing Jia, Xianqun Fan

**Affiliations:** 1 Department of Ophthalmology, Ninth People’s Hospital, Shanghai JiaoTong University School of Medicine, Shanghai, China; 2 Shanghai Key Laboratory of Orbital Diseases and Ocular Oncology, Shanghai, China; 3 Shanghai Institute of Immunology, Shanghai JiaoTong University School of Medicine, Shanghai, People’s Republic of China

**Keywords:** miR-181, Uveal melanoma, CTDSPL, E2F1, Cell cycle

## Abstract

**Background:**

MicroRNAs (miRNAs) have been shown to function in many different cellular processes, including proliferation, apoptosis, differentiation and development. *miR-181a*, -*181b*, *-181c* and *-181d* are *miR-181* members of the family, which has been rarely studied, especially uveal melanoma.

**Methods:**

The expression level of miR-181 family in human uveal melanoma cell lines was measured via real-time PCR (RT-PCR). The function of miR-181 on cell cycle was detected through Flow Cytometry assay. Microarray assay and Bioinformatics analysis were used to find the potential target of miR-181b, and dual-luciferase reporter assays further identified the target gene.

**Results:**

*MiR-181* family members were found to be highly homologous across different species and their upregulation significantly induces UM cell cycle progression. Of the family members, *miR-181b* was significantly overexpressed in UM tissues and most UM cells. Bioinformatics and dual luciferase reporter assay confirmed CTDSPL as a target of *miR-181b*. *miR-181b* over-expression inhibited CTDSPL expression, which in turn led to the phosphorylation of RB and an accumulation of the downstream cell cycle effector E2F1, promoting cell cycle progression in UM cells. Knockdown CTDSPL using siRNAs showing the same effect, including increase of E2F1 and the progression of cell cycle.

**Conclusions:**

*MiR-181* family members are key negative regulators of CTDSPL-mediated cell cycle progression. These results highlight that *miR-181* family members, especially *miR-181b*, may be useful in the development of miRNA-based therapies and may serve as novel diagnostic and therapeutic candidate for UM.

**Electronic supplementary material:**

The online version of this article (10.1186/s13046-018-0679-5) contains supplementary material, which is available to authorized users.

## Background

Recently, miRNAs were found to play critical roles in many different cellular processes, especially in tumor progression. There are nearly 1000 miRNAs and more than 40,000 protein-coding genes in the human genome [1, 2]. Consequently, it is more feasible to explore reliable miRNA biomarkers from genome-wide miRNA expression data than from genome-wide gene expression data. miRNAs are a class of short non-coding RNAs ranging from 19 to 25 nucleotides in length, which are transcribed as precursors and are matured to active forms by a series of enzymes, including Dicer [3]. Numerous studies have explored the instrumental roles of these small, non-coding RNA species, mostly through base-pairing to the untranslated region (UTR) of target mRNA, thus leading to its degradation and/or reduced translation [4]. Generally, an individual miRNA can regulate the expression of multiple target genes, and several miRNAs can synergistically act on one target gene, regulating cell differentiation, proliferation, mobility and apoptosis [5]. The *miR-181* family contains four miRNAs (*miR-181a/b/c/d*). *miR-181a* and *miR-181b* are transcribed from two separated gene loci (*miR-181a-1/miR-181b-1* and *miR-181a-2/miR-181b-2*), while *miR-181c* and *miR-181d* are transcribed from another locus [6]. It had been reported that *miR-181a*, *-181b*, *-181c* and *-181d* function differently in a tumor series. However, the homology among the miR-181 family members and the contribution of *miR-181a*, *-181b*, *-181c* and *-181d* in UM have not yet been clarified.

UM is the most frequent malignant intraocular cancer in adults, and up to 50% of UM patients are at risk of metastasis via hematogenous spread, most commonly to the liver [7]. Recently, epigenetic events mediated by miRNAs have been implicated in UM development. UM proliferation and progression are regulated by dynamic interactions between UM-specific regulators, including miRNAs, whose aberrant expression has been associated with oncogenesis and tumor suppressor activity [8]. Recent studies have implicated miRNAs in UM development. For example, *miR-20a* functions as an oncogenic miRNA involved in promoting cell growth in UM, and *miR-454* promotes proliferation and invasion by regulating *PTEN* in UM [9, 10]. On the other hand, *miR-32* and *miR-124a* both function as tumor suppressors by regulating multiple targets involved in UM development [11, 12]. Moreover, growing evidence indicates that miRNA expression can potentially be used as a biomarker for the diagnosis and prognosis of different tumors. However, the expression and function of the *miR-181* family members in the pathogenesis of UM had not been established.

In the present study, the homology and function of *miR-181* family members, *miR-181a*, *-181b*, *-181c*, and *-181d*, were investigated. *miR-181* family members were found to be highly homologous and have the same target, *CTDSPL*. The CTDSPL gene contains 8 exons coding for a 4.8 kb mRNA, which has been previously denoted as HYA22 and RBSP3, is a recently identified phosphatase-like tumor suppressor gene that dephosphorylates the Rb1 serine on Ser-807 and Ser-811 [13]. The sequence analysis shows that CTDSPL belongs to a gene family of small C-terminal domain phosphatases that may control the RNA polymerase II transcription machinery [14]. Then, the pattern of miRNA expression in melanoma tissues was analyzed using microarray technology. The microarray results indicated *miR-181b1* and *miR-181b2* were highly expressed in melanoma tissues. Furthermore, *miR-181b* was found to be extremely overexpressed in most UM cells. These findings raised the possibility that *miR-181b* might have an important role in UM development or pathogenesis. However, the molecular basis for this phenotype has not been elucidated, and the status of the downstream targets of *miR-181b* in UM has not been researched. Therefore, a better understanding of the mechanisms responsible for UM and an exploration of the novel diagnostic and therapeutic strategies are crucial for achieving improved patient outcomes.

## Methods

### Cell culture and transfection

UM cells SP6.5, VUP, OCM1 and 92-1 were maintained in Dulbecco’s Modified Essential Medium (DMEM; Gibco, Carlsbad, CA, USA) with 10% fetal bovine serum (FBS; Gibco) OCM1a and MUM2b were maintained in Iscove’s Modified Dulbecco’s Medium (IMDM; Gibco) with 10% FBS. The normal control cells, RPE, were maintained in DMEM with 10% FBS. Cultures were maintained at 37 °C in a 5% CO_2_ humidified atmosphere. Cells were treated and harvested for qRT-PCR and Western blot analysis. MUM2b (3 × 10^5^) or OCM1a (5 × 10^5^) cells were cultured overnight in 6-well plates and transfected with 200 nM *miR-NC*, *miR-181* family mimics, or as-*miR-181* family members (GenePharma Co. Ltd., Shanghai, China) using Lipofectamine 2000 (Invitrogen, Carlsbad, CA, USA). Two days later, these cells were either harvested for protein and mRNA or fixed using 70% ethanol for FCM.

### Cell cycle analysis

Treated UM cells along with control cells were harvested. The cells were washed twice with cold phosphate-buffered saline (PBS), fixed in 70% ethanol and stored at 4 °C overnight. The next day, the cells were washed twice with cold PBS and incubated with propidium iodide/ribonuclease staining solution (Becton Dickinson, NJ, USA) for 15 min at room temperature, following the manufacturer’s instructions. Cell cycle distribution was detected and analyzed using the FACScan instrument and CellQuest program (Becton Dickinson, NJ, USA).

### Western blot analysis

After the indicated treatments, the cells were washed with PBS and lysed with ice-cold lysis buffer (RIPA; Sigma Chemical Co, MO, USA). Cell lysates were incubated at 4 °C for 50 min. After centrifugation at 12,000 g for 1 min at 4 °C, protein concentration was determined by a BCA protein assay (Bio-Rad, Hercules, CA, USA). Thirty micrograms of protein were separated on 10% SDS–PAGE and transferred to a PVDF membrane. Membranes were probed with primary antibodies against CTDSPL (Abcam, Cambridge, UK) or E2F1 (Abcam, Cambridge, UK) at 4 °C overnight. Next, the membranes were washed three times with TBS containing 0.1% Tween-20 and incubated with secondary antibody for 1 h. The PVDF membrane was washed three times with Tris-Buffered Saline Tween-20 (TBST). After washing with TBST, the bands were detected using the Odyssey Infrared imaging system (Odyssey; LI-COR, Lincoln, NE).

### Dual-luciferase reporter assay

To determine the common target region of the miR-181 family in *CTDSPL*, a segment of wild-type and mutated 3’-UTR of the human *CTDSPL* cDNA was constructed. Constructs were validated by sequencing. 293 T cells were plated in 24-well flat-bottomed plates and co-transfected with the wild-type or mutated 3’-UTR of the *CTDSPL* reporter plasmid, pRL-TK, and *miR*-*181* family members or *miR*-*NC* using Lipofectamine 2000 reagent. Firefly and Renilla luciferase activities were determined 24 h after transfection using the dual-luciferase reporter assay system (Promega, Madison, WI, USA). The Renilla values were normalized to firefly luciferase.

### Microarray and computational analysis

Briefly, RNA from tissue samples (three melanomas and three normal tissues) was used to synthesize double-stranded cDNA, and double-stranded cDNA was labeled and hybridized to the 2.0 microRNA Expression Microarray (Affymetrix GeneChip Human Gene 2.0 ST Array, Rockville, MD, USA). Raw data were extracted as pair files using NimbleScan software (version 2.5; Roche NimbleGen, Inc., Madison, WI, USA). NimbleScan software’s implementation of RMA offers quartile normalization and background correction. Differentially expressed genes were identified through the random variance model. The AP value was calculated using the paired t-test. The threshold set for up- and downregulated genes was a fold change > 2.0 and a *P*-value < 0.05. Hierarchical clustering was performed based on differentially expressed miRNAs using Cluster_Treeview software from Stanford University (Palo Alto, CA, USA).

### RNA extraction, reverse transcription and quantitative polymerase chain reaction (RT-qPCR)

Total RNA from UM cells was isolated using the Trizol reagent (Invitrogen, Carlsbad, CA, USA) following the protocol provided by the manufacturer. The cDNA synthesis reaction was performed according to the manufacturer’s protocol (TaKaRa Bio, Otsu, Japan). DPN1 enzyme (Sangon Biotech, Shanghai, China) was used to delete the genomic DNA from the extracted RNA, which was used to amplify the miRNAs. Screening for miRNAs was performed by qRT-PCR with the primer sets described in Table 1. PCR reactions were performed according to the manufacturer’s protocol (TaKaRa Bio) and were repeated at least three times for each sample. The miRNA loop primers were used first, and then the miRNAs PCR primers used. The relative levels of target gene miRNA transcripts to control *U6* were determined by the 2^-△△CT^ method.

**Table 1:** Primers used in this study

Primer name	Sequence (5′- 3′)
*hsa-miR-181a-5p loop*	GTCGTATCCAGTGCAGGGTCCGAGGTATTCGCACTGGATACGACACTCACCG
*hsa-miR-181a-5p F*	TGCGCAACATTCAACGCTGTCG
*hsa-miR-181a-5p R*	CTCAAGTGTCGTGGAGTCGGCAA
*hsa-miR-181b-5p loop*	GTCGTATCCAGTGCAGGGTCCGAGGTATTCGCACTGGATACGACACCCACCG
*hsa-miR-181b-5p F*	TGCGCAACATTCATTGCTGTCG
*hsa-miR-181b-5p R*	CTCAAGTGTCGTGGAGTCGGCAA
*hsa-miR-181c-5p loop*	GTCGTATCCAGTGCAGGGTCCGAGGTATTCGCACTGGATACGACACTCACCG
*hsa-miR-181c-5p F*	TGCGCAACATTCAACCTGTCG
*hsa-miR-181c-5p R*	CTCAAGTGTCGTGGAGTCGGCAA
*hsa-miR-181d-5p loop*	GTCGTATCCAGTGCAGGGTCCGAGGTATTCGCACTGGATACGACACCCACCG
*hsa-miR-181d-5p F*	TGCGCAACATTCATTGTTGTCG
*hsa-miR-181d-5p R*	CTCAAGTGTCGTGGAGTCGGCAA
*U6 F*	CGCTTCGGCAGCACATATAC
*U6 R*	AAATATGGAACGCTTCACGA
*CTDSPL-F*	GTGGCTGACCTCCTAGACC
*CTDSPL-R*	TTCACGTAGTTCCCACGATGA
*GAPDH-F*	GGCTGTTGTCATACTTCTCATGG
*GAPDH-R*	GGCTGTTGTCATACTTCTCATGG
*CTDSPL-si1*	GCAGCAUCCUUAGCUCCUUTT
*CTDSPL-si2*	UCCACCAGCUAAGUACCUUTT

### Overexpressing miR-181b plasmid construction, lentivirus package, cloning and stable transfection in UM cells

The *miR-181b* sequence was amplified and sequenced without mutations. Then, 293 T cells were used to package the lentivirus. 293 T cells were cultured in DMEM supplemented with 10% FBS and maintained at 37 °C at a concentration of 6 × 10^6^ cells/ml and transfected using Lipofectamine 2000 reagent with 3 μg PL-shRNA-HSA-MIR-181b-5p, 3 μg pMD2.D, and 6.0 μg PsPax. After incubation overnight with the 293 T cells, the media was replaced with 5 mL of fresh medium. The viral supernatants were concentrated and used to obtain stably transfected *miR-181b*-overexpressing UM cells. Stable MUM2b and OCM1a cell lines were established by lentiviral infection and blasticidin selection. The colonies with GFP expression were selected for subsequent culture after incubation with 4 g/mL blasticidin for 3 weeks. Transduction efficiency was determined by EGFP expression and measured by qRT-PCR.

### Statistical analysis

All experiments were carried out in triplicate. All statistical analyses were performed using SPSS19.0 software. The statistical analysis was performed with a double-sided Student’s *t*-test for comparison of two groups. All data are expressed as the mean ± standard error of the mean (SEM). Differences at *P* < 0.05 were considered statistically significant.

## Results

### *miR-181* family members are highly conserved, and their upregulation promotes cell cycle progression

**Fig. 1:**
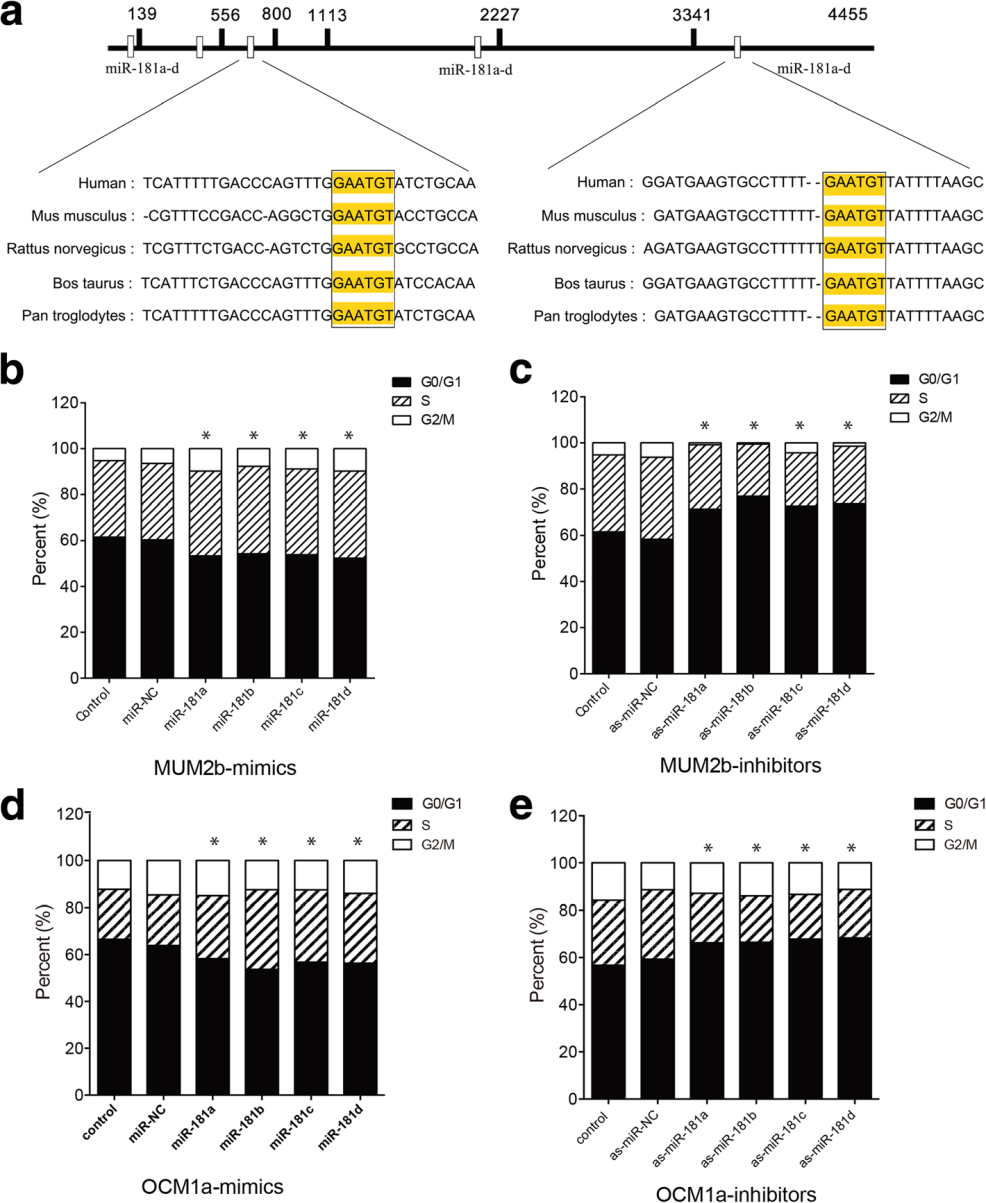
The conservation and cell cycle analysis of *miR-181* family members. **a** Schematic of the *miR*-*181* family putative target sites in the human 3’-UTR of *CTDSPL*. The sequences of the *miR*-*181* family members are partly evolutionarily conserved in *Homo sapiens*, *Mus musculus, Rattus norvegicus, Bos taurus* and *Pan troglodytes*. Yellow indicates the conserved sequence. The hollow white rectangle indicates the five different gene loci of the *miR-181* family members. **b** and (**c**) The cell cycle distribution was detected. The fraction of cells in G0/G1-phase was significantly decreased by 8-12%, and the periods of S-phases were significantly increased 6-15% after the mimics of *miR*-*181* family members were transfected into MUM2b cells compared with the control and miR-NC groups (*P* < 0.05). In contrast, the fraction of cells in G0/G1 phase was significantly increased by 15-18%, and the period of S-phases was significantly decreased 15-20% after the inhibitors of *miR*-*181* family members were transfected into MUM2b cells. **d** and (**e**) The fraction of cells in G0/G1-phase was significantly decreased by 10-20%, and the periods of S-phases were significantly increased 8-18% after the mimics of *miR*-*181* family members were transfected into OCM1a cells compared with the control and miR-NC groups (*P* < 0.05). In contrast, the fraction of cells in G0/G1 phase was significantly increased by 12-15%, and the period of S-phases was significantly decreased 8-12% after the inhibitors of *miR*-*181* family members were transfected into OCM1a cells

To explore the relationship among *miR-181* family members, their sequence homology was investigated. Evolutionary conservation analysis of the *miR-181* family members indicated that the sequences of *miR-181a*, *-181b*, *-181c*, and *-181d* are partly conserved in *Homo sapiens*, *Mus musculus, Rattus norvegicus, Bos taurus* and *Pan troglodytes* (Fig. 1a). To investigate the potential roles of *miR-181* family members, miR-181 family mimics (*miR-181a*, *-181b*, *-181c*, and *-181d*) or inhibitors (*as*-*miR-181a*, *-181b*, *-181c*, and *-181d*) were separately transfected into MUM2b and OCM1a cells. The results demonstrated that mimics of *miR-181* family members promoted cell cycle progression, while inhibitors of *miR-181* family members led to cell cycle arrest (Fig. 1b-e).

### Bioinformatics and molecular biology assays confirmed CTDSPL as a target of *miR-181* family members

**Fig. 2:**
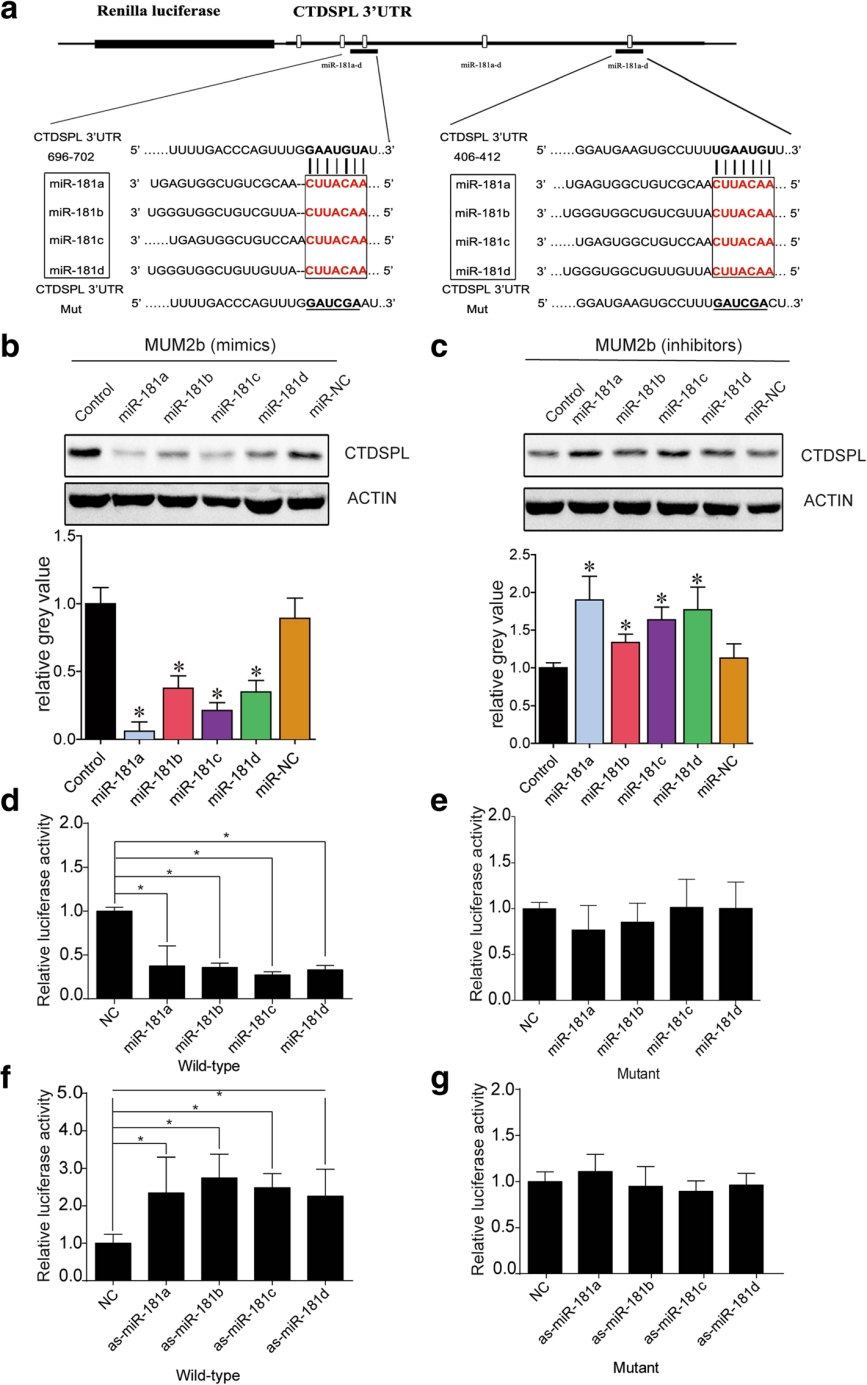
CTDSPL is a direct target of miR-181 family members. **a** Alignment of the seed sites in the human 3’-UTR of *CTDSPL* gene. The mutated 3’-UTR of *CTDSPL* is underlined. **b** and (**c**) MUM2b cells were transfected with 40 nM *miR-NC*, *miR*-*181a*, -*181b*, -*181c*, and -*181d* or *as-miR*-*181a*, -*181b*, -*181c* and -*181d*. Overexpression or knockdown of *miR*-*181* family inhibited or enhanced CTDSPL expression, respectively. The gray level was analyzed by histogram. **d** and (**f**) Overexpression or knockdown of *miR-181* expression inhibited or enhanced the Renilla luciferase activity, respectively. 293 T cells were cotransfected with 40 nM *miR*-*NC*, *miR*-*181a*, -*181b*, -*181c*, and -*181d*, or *as*-*miR*-*181a*, -*181b*, -*181c*, and -*181d*, and 100 ng of reporter plasmid containing the wild-type 3’-UTR of *CTDSPL*. After 24 h, Renilla luciferase values, normalized against firefly luciferase, were measured. **e** and (**g**) The Renilla luciferase activity was nearly unchanged after mimics and inhibitors of *miR*-*181* family members were transfected with the mutated 3’-UTR of *CTDSPL*

To identify the target gene(s) of *miR-181*, candidate genes were identified using the miRNA target prediction database TargetScan [13] (http://www.targetscan.org/), miRanda [14] (http://www.microrna.org/) and Pictar [15] (http://pictar.mdc-berlin.de/). miR-181 family members were predicted to target *CTDSPL*, which had previously been denoted as *RBSP3* (*RB1* serine phosphatase from human chromosome 3), a key downstream mediator of cell cycle progression, and has been reported to participate in acute myeloid leukemia pathogenesis [6]. There are five predicted target sites in the 3’-UTR of *CTDSPL* sequence for *miR-181* family members. The predicted sequences to which *miR-181* binds in the 3’-UTR of *CTDSPL* are conserved in humans (Fig. 2a). Western blot assays further indicated that mimics of *miR-181* family members led to the reduced expression of *CTDSPL*, while inhibitors led to the increased expression of CTDSPL in MUM2b cells (Fig. 2b-c, *P* < 0.05). To examine whether *miR-181* family members could directly regulate CTDSPL expression, 293 T cells were transfected with a luciferase reporter construct containing the putative wild-type and mutant 3’-UTR of CTDSPL binding sites, together with one of the following miRNAs: *miR-181a*, *-181b*, *-181c*, *-181d*, *miR-NC*, *as-miR-181a*, -*181b*, -*181c*, or -*181d*. Compared with control cells, firefly luciferase activity was significantly decreased by nearly 3-fold after treatment with *miR-181a*, *-181b*, *-181c*, and *-181d* mimics and cotransfection with the wild-type *CTDSPL* gene 3’-UTR plasmid (Fig. 2d), whereas activity increased approximately 3-fold when *miR-181a*, *-181b*, *-181c*, or *-181d* inhibitors were used (Fig. 2f). Firefly luciferase activity was largely unchanged when the putative mutant of the 3’-UTR of *CTDSPL* binding sites was used (Fig. 2e and g). These data provide strong evidence that the *miR-181* family members inhibit *CTDSPL* gene expression by directly binding to sites within its 3’-UTR.

### *miR-181b* was extremely overexpressed in melanoma tissues and most UM cells

**Fig. 3:**
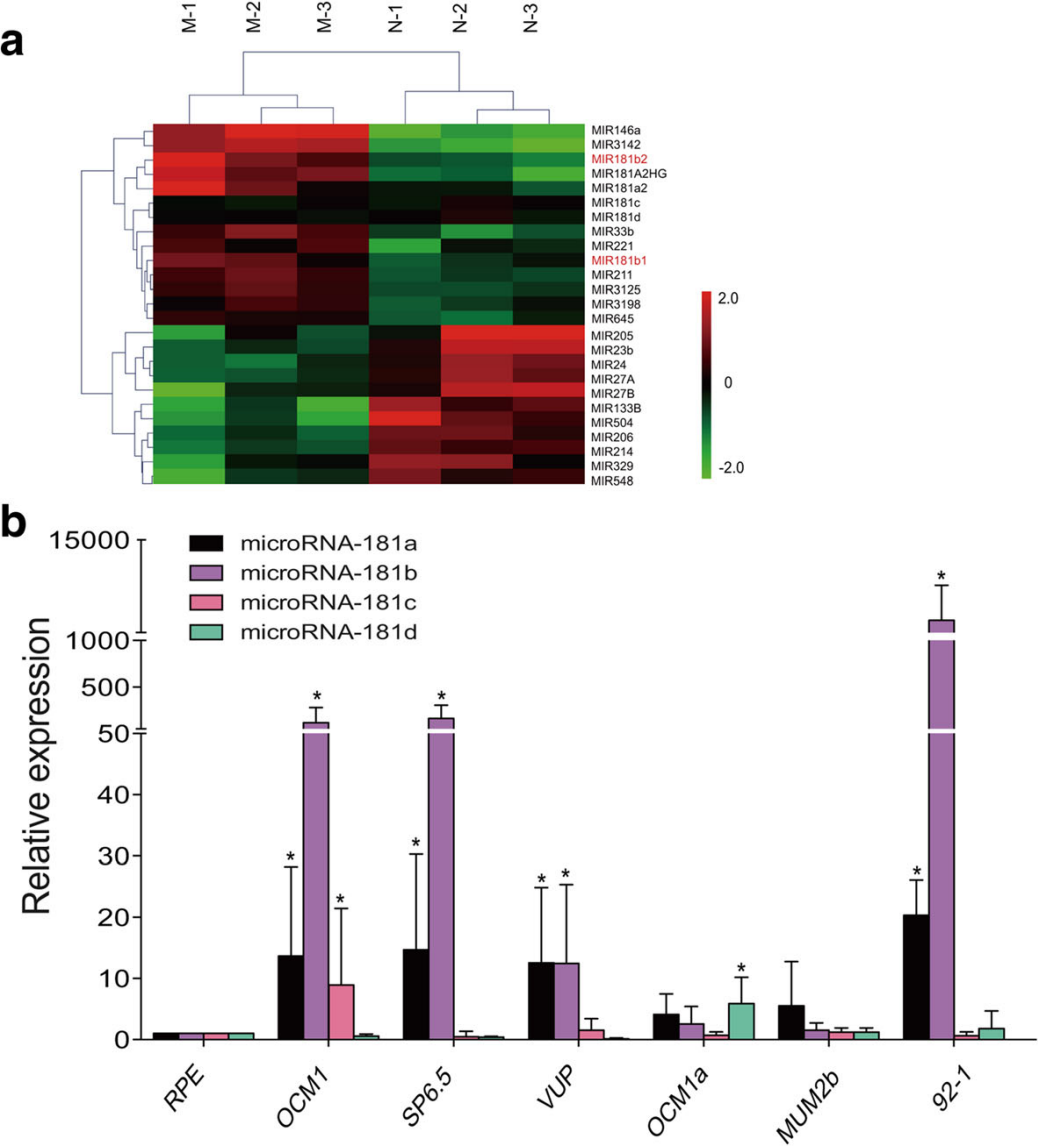
The expression profile of *miR*-*181* in melanoma tissues and UM cells. **a** Hierarchical clustering analysis of miRNAs that were differentially expressed in melanoma compared with non-tumor samples. Expression values are represented in shades of red and green indicating expression above and below the median expression value across all samples (log scale 2, from − 2 to + 2), respectively. *miR-181b1* and *miR-181b2* were significantly upregulated in melanoma tissues. **b** The expression of *miRNA-181a-d* was measured by qRT-PCR in RPE, OCM1, SP6.5, VUP, OCM1a, MUM2b and 92-1 cells. *miR*-*181b* was overexpressed in OCM1, SP6.5, VUP and 92-1 cells by approximately 50-fold and more than 1000-fold in 92-1 cells, while *miR*-*181b* was not upregulated in OCM1a or MUM2b cells. *miR*-*181a* was upregulated in OCM1, SP6.5, VUP and 92-1 cells by approximately 12-to-20-fold. The expression levels of *miR-181c* and *miR*-*181d* were not upregulated in most UM cell lines, except for a slight increase in *miR*-*181c* in OCM1 cells and *miR*-*181d* in OCM1a cells, both less than 10-fold. There was no downregulation of any *miR*-*181* family members. Triplicate assays were performed for each sample, and the relative level of each miRNA was normalized to *U6* (*P < 0.05)

To investigate the expression profile of *miR-181* family members in UM, microarray technology was used to detect the expression of *miR-181* family members in melanoma tissues. Compared with normal samples, gene chip results showed that *miR-181b1* and *miR-181b2* were significantly overexpressed in melanoma tissues. *miR-181a* expression was also upregulated, while *miR-181c* and *miR-181d* were essentially unchanged (Fig. 3a). Next, *miR-181* family members were detected in various types of UM cells, including OCM1, SP6.5, VUP, OCM1a, MUM2b and 92-1 cells. In accordance with the microarray results, miR-181b was overexpressed in OCM1, SP6.5, VUP and 92-1 cells by nearly 50-fold and was up more than 1000-fold in 92-1 cells, while *miR-181b* was not upregulated in OCM1a or MUM2b cells (Fig. 3b). miR-181a was also upregulated in OCM1, SP6.5, VUP and 92-1 cells 12-20-fold. The expression levels of *miR-181c* and *miR-181d* were not upregulated in most UM cell lines, except for a slight increase in *miR-181c* in OCM1 cells and a mild upregulation of *miR-181d* in OCM1a, both less than 10-fold. Additionally, there was no downregulation of any *miR*-*181* family member (Fig. 3b) in these cell lines. These results show that *miR*-*181b* exhibited significantly higher expression in most UM cells, strongly implying a relationship between the upregulation of *miR*-*181b* and UM development and that *miR*-*181b* expression is a specific marker of UM.

### *miR-181b* overexpression promotes cell cycle progression through CTDSPL with the downstream release of E2F1 in MUM2b and OCM1a cells

**Fig. 4:**
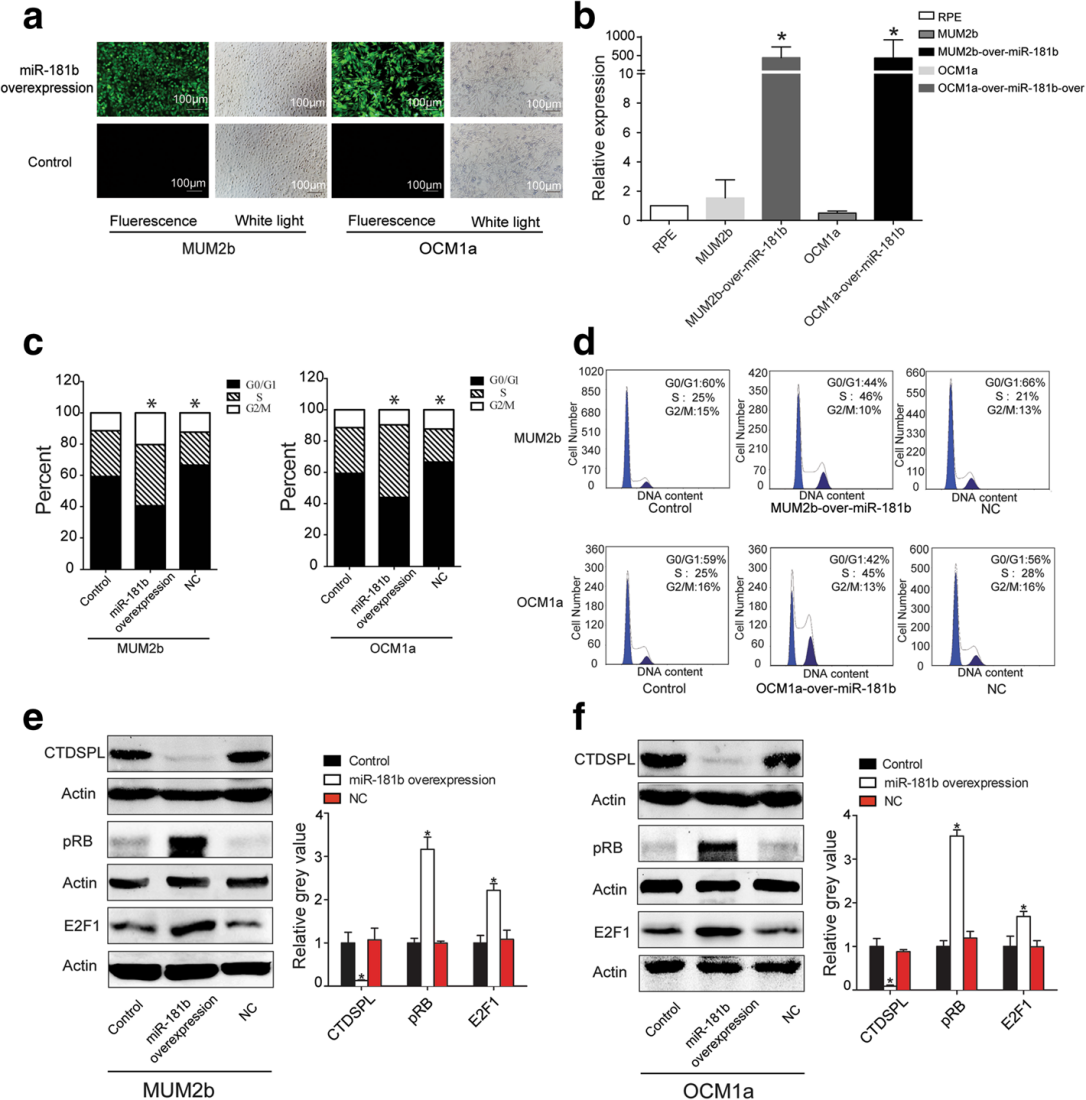
*miR*-*181b* inhibits cell cycle distribution through CTDSPL and E2F1. **a** The *miR*-*181b* overexpression plasmid was stably transfected into MUM2b and OCM1a cells (MUM2b-over-*miR*-*181b*, OCM1a-over-*miR*-*181b*), and the plasmid also contained the EGFP tag. Obvious green fluorescence was observed in MUM2b-over-miR-181b and OCM1a-over-miR-181b cells, but not in the control groups (original magnification 100X). **b** The qRT-PCR results showed significantly higher expression of miR-181b of nearly 500-fold in MUM2b-over-*miR*-*181b* and OCM1a-over-*miR*-*181b* cells. **c** and (**d**) Cell cycle progression was significantly promoted in *miR*-*181b*-stably transfected MUM2b and OCM1a cells. The G0/G1 phase was significantly decreased by approximately 19% and 16%, while that the S-phases was significantly increased by about 10% and 18% in MUM2b-over-miR-181b and OCM1a-over-*miR*-*181b* cells, respectively, compared with the control group. **e** CTDSPL expression was significantly inhibited in *miR*-*181b*-stably transfected MUM2b and OCM1a cells. **f** E2F1 expression was significantly overexpressed in *miR*-*181b*-transfected MUM2b and OCM1a cells (*P < 0.05)

To explore the function of *miR-181b*, we constructed a high-level expression plasmid of human *miR-181b*. Our earlier qRT-PCR results had demonstrated that *miR*-*181b* was highly expressed in a variety of UM cells, except MUM2b and OCM1a cells. Thus, MUM2b and OCM1a cells were stably transfected with a *miR*-*181b* overexpression plasmid (MUM2b-over-*miR*-*181b*, OCM1a-over-*miR*-*181b*), which also contained an EGFP tag. The transfection efficiency of the stably transfected MUM2b and OCM1a cells was determined by immunofluorescence and qRT–PCR (Fig. 4a-b). There were high amounts of green fluorescence in both the MUM2b-over-*miR*-*181b* and OCM1a-over-*miR*-*181b* cells. However, green fluorescence was not observed in the control cells (Fig. 4a). The qRT-PCR results showed a significantly higher expression of *miR*-*181b* with a nearly 500-fold increase in both the MUM2b-over-*miR*-*181b* and OCM1a-over-*miR*-*181b* cells (Fig. 4b, *P* < 0.05). Next, the cell cycle distribution was analyzed to assess whether *miR*-*181b* affected the cell cycle of MUM2b or OCM1a cells. We found that cell cycle progression was promoted in both the MUM2b-over-*miR*-*181b* and OCM1a-over-*miR*-*181b* cells. The fraction of cells in G0/G1-phase was significantly decreased by approximately 19% and 16%, while that the S-phases was significantly increased by about 10% and 18% in MUM2b-over-*miR*-*181b* and OCM1a-over-*miR*-*181b* cells, respectively, compared with the control group (Fig. 4c-d, P < 0.05). These results suggest that *miR*-*181b* might act as a regulator of UM cell cycle progression. To explore whether CTDSPL is involved in the UM cell cycle, we determined the expression level of CTDSPL protein in UM cells. A decreased expression in CTDSPL and an increased expression of E2F1 were found in MUM2b-over-*miR*-*181b* and OCM1a-over-*miR*-*181b* cells compared with the control group (Fig. 4e-f).

### Decreased CTDSPL expression promotes cell cycle progression in MUM2b and OCM1a cells

**Fig. 5:**
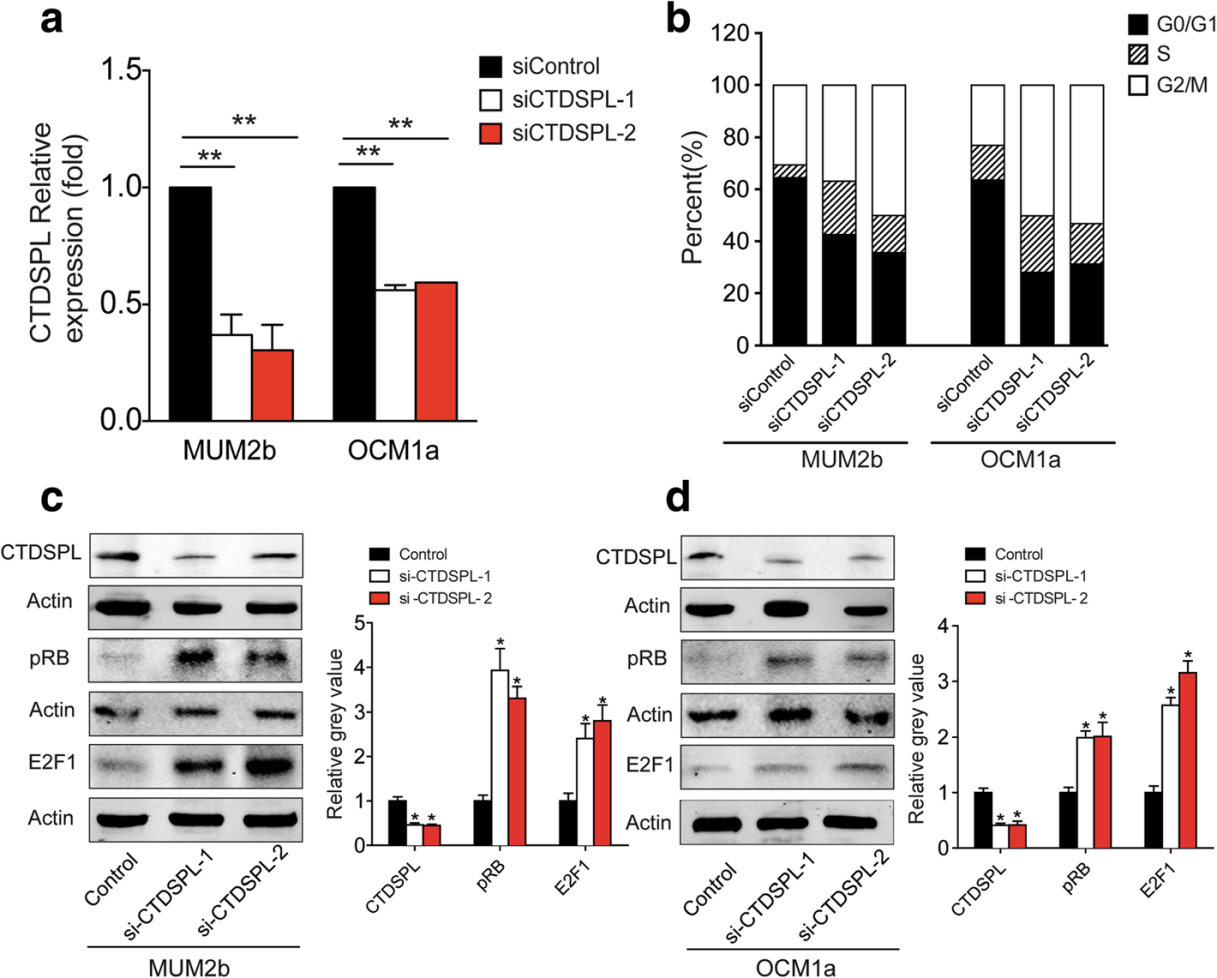
Decreased *CTDSPL* expression promotes cell cycle distribution through pRB and E2F1. **a** qRT-PCR results showed significantly decreased expression of CTDSPL after transfection with si-CTDSPL-1 and si-CTDSPL-2 in MUM2b and OCM1a cells, respectively. **b** The cell cycle G0/G1-phase proportion decreased significantly, approximately 43% and 36% for MUM2b cells and 35% and 32% for OCM1a cells after transfection with si-CTDSPL-1 and si-CTDSPL-2, compared with control group which is 65% in MUM2b cells and 64% in OCM1a cells. While the cell cycle S-phase proportion increased from 13% to 21% and 15% in MUM2b cells and from 10% to 22% and 15% in OCM1a cells after transfection with si-CTDSPL-1 and si-CTDSPL-2 seperately (Fig. 5b). **c-d** CTDSPL expression was significantly inhibited after transfection with si-CTDSPL-1 and si-CTDSPL-2 in MUM2b and OCM1a cells, whereas pRB and E2F1 expression was significantly increased in MUM2b and OCM1a cells after transfection with si-CTDSPL-1 and si-CTDSPL-2. The gray level was analyzed by histogram seperately (*P < 0.05)

To detect the function of CTDSPL, we inhibited expression of CTDSPL in MUM2b and OCM1a cells via transfection of CTDSPL inhibitors (si-CTDSPL-1 and si-CTDSPL-2). Results of qRT-PCR demonstrated that CTDSPL expression was inhibited in UM cells after transfection of si-CTDSPL-1 and si-CTDSPL-2, respectively, approximately 65% and 75% in MUM2b cells and approximately 40% and 35% in OCM1a cells (Fig. 5a). Western blotting further confirmed that expression of CTDSPL decreased in MUM2b and OCM1a cells after transfection with si-CTDSPL-1 and si-CTDSPL-2 (Fig. 5c-d). In addition, the cell cycle G0/G1-phase proportion decreased significantly, approximately 43% and 36% for MUM2b cells and 35% and 32% for OCM1a cells after transfection with si-CTDSPL-1 and si-CTDSPL-2, compared with control group which is 65% in MUM2b cells and 64% in OCM1a cells. While the cell cycle S-phase proportion increased from 13% to 21% and 15% in MUM2b cells and from 10% to 22% and 15% in OCM1a cells after transfection with si-CTDSPL-1 and si-CTDSPL-2 seperately (Fig. 5b). Increased pRB and E2F1 expression was also confirmed after transfection with si-CTDSPL-1 and si-CTDSPL-2 (Fig. 5c-d).

### *miR-181* contributes to cell cycle progression via its target CTDSPL, which in turn increases expression of the cell cycle effector pRB/E2F1 in UM cells

**Fig. 6:**
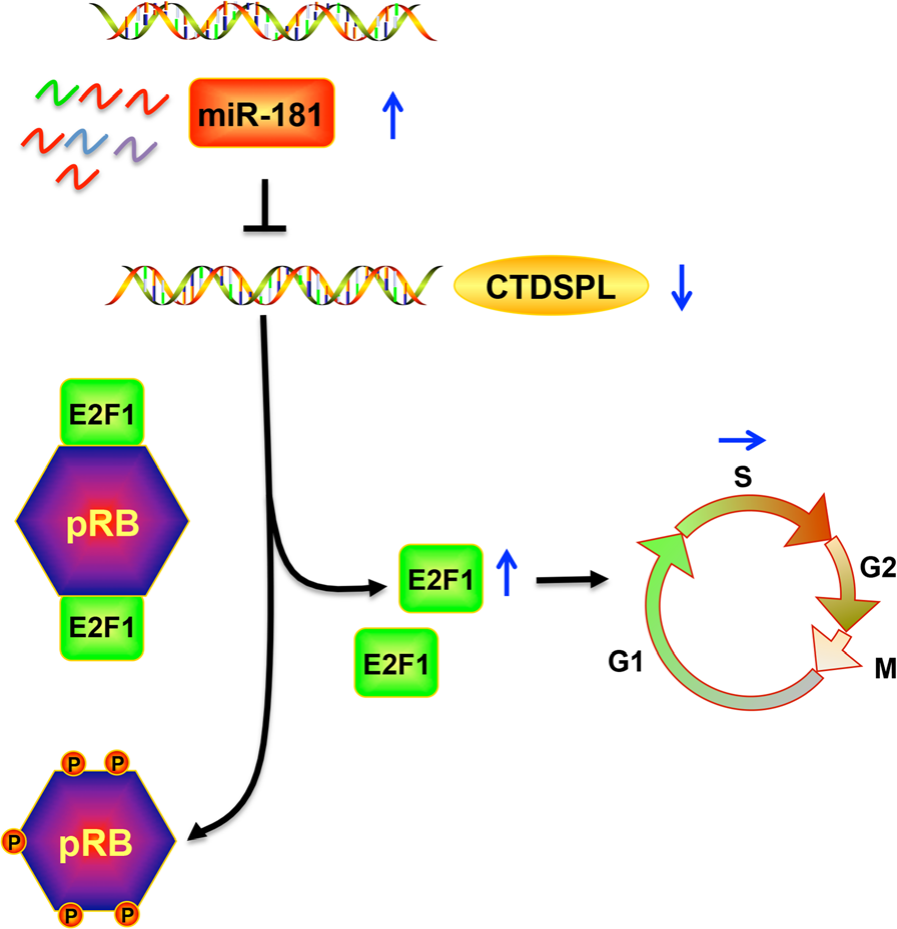
*miR-181* targets *CTDSPL*, which modulates the cell cycle effector E2F1. Schematic representation of the pathway modulated by *miR-181* in UM cells progressing through the cell cycle. *miR-181* overexpression in UM cells induces progression through the G1/S transition and promotes S-phase entry. Thus, miR-181 induces cell cycle progression by repressing the downstream target *CTDSPL*, which in turn results in the phosphorylation of RB and an accumulation of the downstream cell cycle effector E2F1

Our work found that *miR-181b* is overexpressed in melanoma tissues and most UM cells and promotes cell cycle progression by repressing CTDSPL expression in UM cells. Previous studies have revealed that CTDSPL is involved in the regulation of the cell cycle through pRB-E2F1 [13, 15], which removes the phosphate group from serine 807 and 811 in its substrate, phosphorylated RB (pRB), and induces the release of E2F1 protein that then contributes to cell cycle progression [16, 17]. We thus hypothesized that miR-181b might contribute to UM pathogenesis via the CTDSPL–pRB–E2F1 pathway. The decreased CTDSPL expression along with the increased E2F1 expression in MUM2b-over-*miR-181b* and OCM1a-over-*miR*-*181b* cells supports our hypothesis. Collectively, these results demonstrate that *miR*-*181b* over-expression contributes to UM pathogenesis by targeting the CTDSPL–pRB–E2F1 pathway to alter cell cycle progression (Fig. 6).

## Discussion

Recently, miRNAs have emerged as important cellular regulators that mediate cellular proliferation and progression. The *miR*-*181* family consists of *miR*-*181a*, *miR*-*181b*, *miR*-*181c*, and *miR*-*181d*, and *miR*-*181b*, which divided into *miR*-*181b1* and *miR*-*181b2* and is transcribed from two separate gene loci [6]. However, there are few studies on the correlations among *miR*-*181* family members. Here, we explored the correlations among *miR*-*181* family members. First, we found that these miRs are highly evolutionarily conserved in different species, including *Homo sapiens*, *Mus musculus, Rattus norvegicus, Bos taurus* and *Pan troglodytes* (Fig. 1a), which suggests functional conservation. Cell cycle experiments demonstrated that pretreatment with *miR*-*181* family mimics promoted cell cycle progression, while inhibitors resulted in cell cycle arrest (Fig. 1b). Similar effects usually shared the same mechanism and we know that small, non-coding RNA species mostly function through base-pairing to the untranslated region (UTR) of its target mRNA, leading to its degradation and/or reduced translation [18]. To explore the mechanism of the *miR*-*181* family members, we performed bioinformatics analysis, and CTDSPL was identified as a candidate target gene of the *miR*-*181* family members through three publicly available algorithms (TargetScan, miRanda and Pictar). Additionally, it had been previously reported and confirmed that *miR*-*181a* can bind to the 3′-UTR of *CTDSPL* mRNA. However, there are no relevant, precise studies regarding *miR*-*181* [6]. Here, we found that all the *miR*-*181* family members have nearly the same binding site within the 3’-UTR of *CTDSPL*. Additionally, we found five different binding sites (Fig. 2a). A firefly luciferase assay indicated that all *miR*-*181* family members could inhibit CTDSPL expression through directly binding to the 3’-UTR of *CTDSPL*. Consequently, this work confirmed that the *miR*-*181* family members are highly conserved and share a common function of direct binding to the 3’-UTR of *CTDSPL*.

The biological functions of the *miR*-*181* family have been discussed in different tumors with different underlying biological processes. *miR*-*181a* was first identified and recognized as a contributor to hematopoietic lineage commitment and differentiation [19, 20]. In breast cancer, *miR*-*181a* could prevent and reverse drug resistance via binding to the 3’-UTR of BCRP [21]. It has been reported that *miR*-*181a/b* (*miR*-*181a* and *miR*-*181b*) suppress the translation of p300/CBP-associated factor (*PCAF*) mRNA, a process relevant to the epigenetic fine-tuning of epithelial inflammatory processes in liver epithelial cells [22]. There is another report that the loss of *miR-181a-1/b-1* dampens the induction of experimental autoimmune encephalomyelitis and reduces basal TCR signaling in peripheral T cells and their migration from lymph nodes to pathogenic sites [23]. *miR*-*181c* inhibits glioblastoma cell invasion, migration and mesenchymal transition by targeting the TGF-beta pathway and is associated with metastatic brain cancer and high-grade osteosarcoma [24–26]. *miR*-*181d* acts as a glioma suppressor by targeting K-ras and Bcl-2 [27]. Compared with *miR*-*181a*, *miR*-*181c* and *miR*-*181d*, *miR*-*181b* was confirmed to be the most effective miRNA of the *miR*-*181* family members [4, 6]. Studies have demonstrated that *miR*-*181b* is overexpressed in several cancers, such as colorectal cancer, acute lymphocytic leukemia (ALL), acute promyelocytic leukemia and hepatocellular carcinoma [28, 29]. Furthermore, *miR*-*181b* expression was found to be strongly associated with clinical response to S-1 in colon cancer patients [30]. On the other hand, reduced *miR*-*181b* expression has been observed in several primary human cancers, including gastric, lung, and prostate cancer, and acute myeloid leukemia and chronic lymphocytic leukemia [28, 31–33]. However, research regarding the *miR*-*181* family is very scarce. They had been observed in glioblastoma, which could reverse mesenchymal transition by targeting *KPNA4* [34]. At present, there are no investigations exploring their functions and mechanisms in UM pathogenesis and development. Here, microarray technology indicated there are different expression profiles of *miR*-*181a*, *miR*-*181b*, *miR*-*181c*, and *miR*-*181d* in UM. *miR*-*181b1* and *miR*-*181b2* were significantly overexpressed in melanoma tissues, while there were no significant changes in *miR*-*181c* and *miR*-*181d*. We also explored the function of these four miRNAs in UM cells. In accordance with the microarray results, we found *miR*-*181b* was extremely upregulated in most UM cell lines and that *miR*-*181a* was also upregulated in most UM cell lines. However, *miR*-*181c* and *miR*-*181d* were not upregulated in most UM cell lines, except for a slight increase of *miR*-*181c* in OCM1 and *miR*-*181d* in OCM1a. Given the different expression patterns of the *miR*-*181* family members, we can assume that they function differently in different UM tissues and cell lines. Interestingly, there was no downregulation of any *miR*-*181* family members in the UM cells compared with the control group. Thus, there is expression specificity of *miR*-*181* family members in UM, but a lack of universality. In our study, *miR*-*181b* was especially interesting. *miR*-*181b* displayed the highest degree of expression in melanoma tissues and UM cell lines, and several previous studies have revealed that *miR*-*181b* is more active [4, 6] and has an intimate relationship with human malignant tumors, including hepatocellular carcinoma, colorectal gastric, lung, and prostate cancer, and ALL, acute myeloid leukemia, and chronic lymphocytic leukemia [28, 29, 31–33].

In our study, *miR*-*181b* was significantly upregulated in most UM cell lines, specifically VUP, SP6.5, OCM1 and 92-1, but was not upregulated in MUM2b or OCM1a cells. We then transfected MUM2b and OCM1a cells with a *miR*-*181b* overexpression plasmid. Compared with the control group, the cell cycle was at a later stage in miR-181b-overexpressing MUM2b and OCM1a cells. Bioinformatic analyses and dual-luciferase reporter assays demonstrated and confirmed that *CTDSPL* was the target of the *miR*-*181* family members. A significant downregulation of CTDSPL and upregulation of E2F1 in MUM2b-over-*miR*-*181b* and OCM1a-over-*miR*-*181b* cells was confirmed. The underlying molecular pathway responsible for the effects of *miR*-*181b* in UM cell survival might control the G0/G1 to S phase transition through the repression of CTDSPL. *CTDSPL* is an important phosphatase-like tumor suppressor gene located at 3p21.3, and belongs to the small C-terminal domain phosphatase family, which modulates the RB/E2F1 signaling pathway and results in cell cycle arrest at the G1/S boundary [13, 35]. Previous studies have also reported that CTDSPL removes the phosphate group from serines 807 and 811 in its substrate, pRB, and thereby induces the formation of the RB/E2F1 complex [36, 37]. It had been reported that *miR-100* regulates myeloid differentiation by targeting *CTDSPL* [38]. However, the involvement of *CTDSPL* in the regulation of cell growth in UM cells has not yet been studied. Our data demonstrated that overexpressed *miR*-*181b* knocked down CTDSPL expression and resulted in an accelerated G1/S transition in UM cells. These results indicate that overexpressed *miR*-*181b* inactivates the phosphatase CTDSPL protein and that this inactivation may be a common step that is required for UM progression.

In this work, *miR*-*181b* was found to control the G0/G1 to S phase transition by repressing CTDSPL and regulating E2F1 expression in most UM cells, except for MUM2b and OCM1a cells. This finding suggests that *miR-181b* expression is specifically increased or unchanged, without downregulation, in all UM cells, but there is lack of universality in UM. There were other reports that CTDSPL can directly bind to Rb just like the mechanism demonstrated by Beniaminov et al. [39, 40]. It showed a new method called surface plasmon resonance (SPR) to detect the direct interaction. However, in our co-immunoprecipitation assay, no interaction between CTDSPL and RB1 was found in OCM1a cell line (Additional file 1). Previous studies showed that cyclic phosphorylation/dephosphorylation of the pRb protein plays an important role in decreasing ppRb levels, ultimately blocks the G1/S progression. In our study, CTDSPL is another important regulator of ppRb level through phosphatase activity [14, 41, 42], showing as another parallel pathway, without relationship exists between cyclins and CTDSPL, at least in OCM1a cell line (Additional file 1).

*miR*-*181* family members have been reported as potential therapeutic targets for myeloid dysplastic syndrome and acute myeloid leukemia [6]. Although *miR*-*181c* and -*181d* mimics and inhibitors could promote and inhibit CTDSPL expression through the same binding site in the *CTDSPL* gene, *miR-181c* and *miR*-*181d* were not upregulated in most UM cells except for a slight increase in miR-181c in OCM1 cells and miR-181d in OCM1a cells. This finding suggests that *miR*-*181c* and *miR*-*181d* do not play leading roles in UM cells but rather that *miR*-*181c* and *181d* have support and backup functions for *miR*-*181b*, through binding to the 3’-UTR of *CTDSPL* and inhibiting its expression. In other words, while one of the *miR*-*181* family members may be the primary functional miRNA in one tumor, the other *miR*-*181* family members may assist it. Finally, *miR*-*181* family members, and especially *miR*-*181b* overexpression, could be used as therapeutic targets for UM. Our results characterize a new role for *miR*-*181* family members. It is regrettable that *miR*-*181* family members were not detected in the limited UM tissues available, except via the microarray chip. Going forward, we hope to detect the expression of *miR*-*181* in additional UM tissues. The prognostic and therapeutic value of *miR*-*181* family members, especially *miR*-*181b*, needs to be confirmed in UM patients and other tumor types in the future. Moreover, *miR*-*181* expression would be ideally detectable in the blood of UM patients, which would be useful in the future for UM patient diagnosis and prognosis.

## Conclusions

In summary, we have presented herein the novel finding that *miR-181b* contributes to cell cycle progression through depressing the expression of CTDSPL, which in turn activates the downstream effector E2F1 and promotes S-phase entry. Furthermore, *miR*-*181c* and -*181d* might support and backup the function of *miR*-*181b* through binding to the 3’-UTR of CTDSPL and inhibit its expression in UM cells. Taken together, these results suggest that a high *miR-181b* expression may play an important role in UM through disrupting cell cycle control, promoting cell proliferation and consequently facilitating the development of UM via CTDSPL. This might thus represent a therapeutic target in UM. These findings constitute a comprehensive foundation for future research on the important role *miR-181* in the developmental pathology of UM. The manipulation of *miR*-*181* family members could be diagnostically and therapeutically relevant for the treatment of UM.

## Additional file

Additional file 1: There are no interactions between CTDSPL and RB1, cyclins and CTDSPL. (TIF 9130 kb)

## Abbreviations

CTDSPL: (CTD small phosphatase-like)
miRNAs: (microRNAs)
*RBSP3*: (*RB1* serine phosphatase from human chromosome 3)
RT-qPCR: (Reverse transcription quantitative polymerase chain reaction)
UM: (Uveal melanoma)
