# Supplementary figures and images for: The miR-181 family promotes cell cycle by targeting CTDSPL, a phosphatase-like tumor suppressor in uveal melanoma
Source: J Exp Clin Cancer Res. 2018 Jan 30;37:15. doi: 10.1186/s13046-018-0679-5 (PMC5791374; doi:10.1186/s13046-018-0679-5)

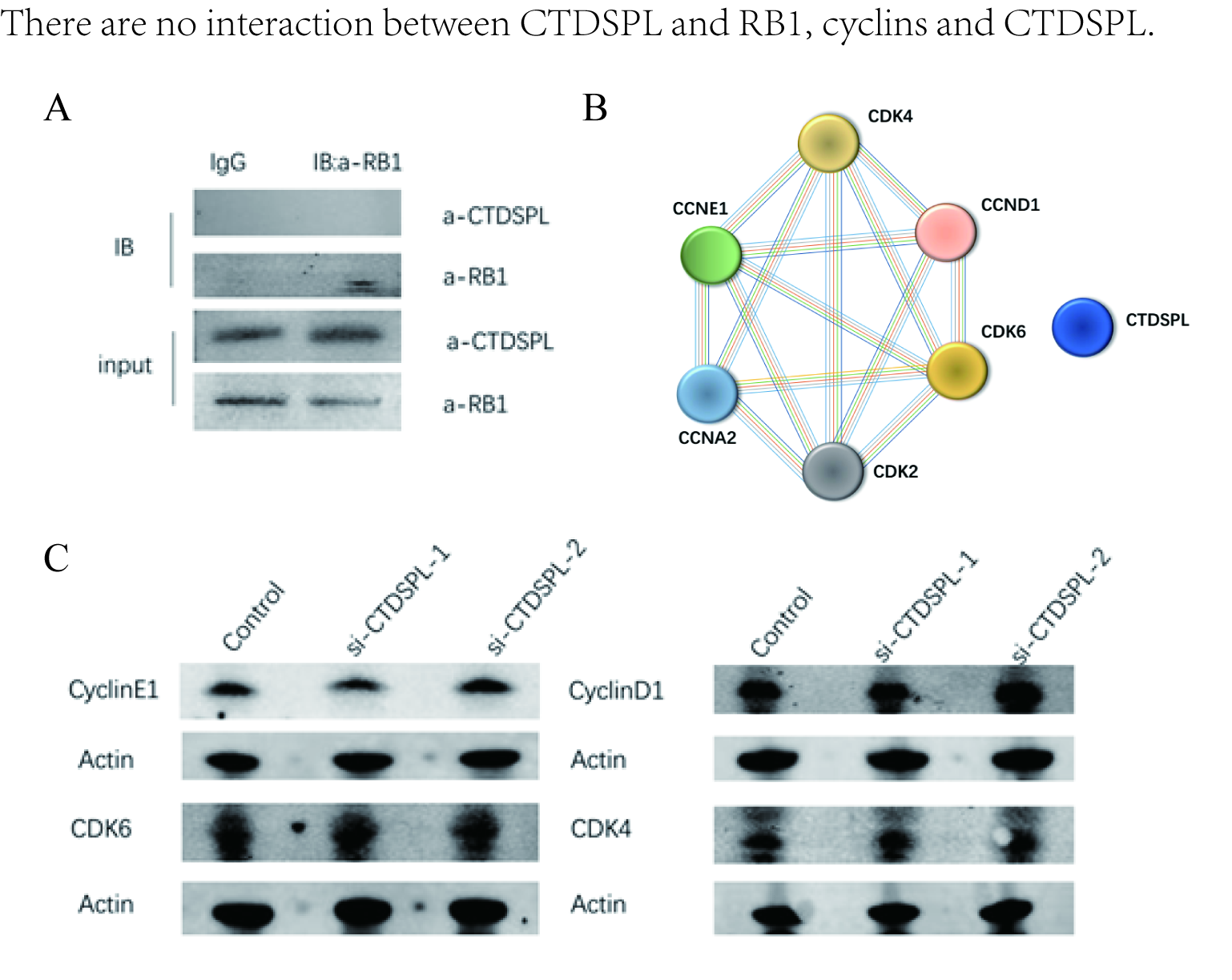

Supplement: Additional file 1: — There are no interactions between CTDSPL and RB1, cyclins and CTDSPL. (TIF 9130 kb) [file 13046_2018_679_MOESM1_ESM.tif]
